# Effectiveness of a community-based intervention for weight loss on cardiometabolic risk factors among overweight and obese women in a low socio-economic urban community: findings of the MyBFF@home

**DOI:** 10.1186/s12905-018-0593-1

**Published:** 2018-07-19

**Authors:** Ahmad Zamri Liyana, Geeta Appannah, Siti Yazmin Zahari Sham, Mansor Fazliana, Noor Safiza Mohamad Nor, Rashidah Ambak, Azah Abdul Samad, Nofi Yuliani Dahlan, Tahir Aris

**Affiliations:** 1 Cardiovascular, Diabetes and Nutrition Research Centre, Institute for Medical Research, National Institutes of Health, Ministry of Health Malaysia, Kuala Lumpur, Malaysia; 2 Department of Nutrition and Dietetics, Faculty of Medicine and Health Sciences, Universiti Putra Malaysia, Serdang, Selangor Malaysia; 3 Department of Pathology, Faculty of Medicine and Health Sciences, Universiti Putra Malaysia, Serdang, Malaysia; 4 Institute for Public Health, National Institutes of Health, Ministry of Health Malaysia, Kuala Lumpur, Malaysia; 5 Shah Alam Seksyen 7 Health Clinic, Persiaran Kayangan, Section 7, Shah Alam, Selangor Malaysia; 6 Occupational Safety & Health Unit, Gombak District of Health Office, Batu Caves, Selangor Malaysia

**Keywords:** Lifestyle intervention, Obesity, Overweight, Cardiometabolic risks, Women

## Abstract

**Background:**

The effectiveness of lifestyle intervention for weight loss on cardiometabolic risk factors among overweight and obese individuals in the community setting remains inconclusive. This study aimed to evaluate the effect of a 6-month weight loss lifestyle intervention on cardiometabolic risk factors among overweight and obese women and the sustainability of the changes in those markers at 12-month follow-up, comparing an intervention group with a control group.

**Methods:**

A total of 243 participants from MyBFF@home were included in this study. Fasting blood samples at baseline, 6- and 12-month were assessed for fasting plasma glucose (FPG), total cholesterol (TC), low-density lipoprotein cholesterol (LDL-C), high-density lipoprotein cholesterol (HDL-C) and triglycerides. The effect of the intervention on cardiometabolic risk markers were investigated within and between study groups using t-test and general linear model (GLM) repeated measure ANOVA.

**Results:**

Results from repeated measures ANOVA showed intervention effect only in TC where significant reduction was found in the intervention group (− 0.26 mmol/L [95% CI: – 0.47 to − 0.06], *p* < 0.01) compared to the control group (− 0.06 mmol/L [95% CI: – 0.28 to 0.17]) at 12 months. At 6 months, TC was reduced significantly in both groups but only intervention group retained the reduction in maintenance phase while, the level increased significantly in the control group (0.22 mmol/L [95% CI: 0.06 to 0.38]). This attributed to significant increase in TC/HDL-C ratio in the control group during maintenance phase (0.32 [95% CI: 0.15 to 0.50], *p* < 0.001). The intervention group also showed trend of reduction in FPG at 6 months and further decreased during maintenance phase (− 0.19 mmol/L [95% CI: – 0.32 to − 0.06], *p* < 0.01). At 6 months HDL-C was maintained in the intervention group but reduced significantly in the control group (− 0.05 mmol/L [95% CI: – 0.10 to − 0.01], *p* < 0.05). No significant difference was detected in both markers when compared between groups.

**Conclusions:**

In the context of low socio-economic communities, this study supports that weight loss related lifestyle modifications over a 6-month period could improve selected cardiometabolic risk factors, particularly fasting glucose, TC and HDL-C in overweight and obese women with favourable sustainability over a 12-month period.

## Background

Globally, the growing obesity epidemic is associated with increased rate of non-communicable diseases (NCD) such as cardiovascular disease (CVD), type-2-diabetes mellitus (T2DM) and cancers. Malaysia is not exceptional from this public health burden whereby the escalating rate of obesity coincided with the increasing prevalence of NCD in the population. Obesity was more prevalent among women in Malaysia compared to their counterparts [1]. Mustafa et al. revealed that 22.1 and 12.6% of Malaysian adults had pre-diabetes and newly diagnosed T2DM with a higher proportion of women in both prevalence (69.8 and 69.0% respectively), and a higher mean body mass index (> 25 kg/m^2^) in both groups [2]. Concurrently increasing sedentary behaviour in the population mainly among women [3] coupled with rapid nutritional transition [4] further exacerbates weight gain in this group.

Lifestyle interventions are shown to be effective to promote modest yet, clinically significant weight reduction and can improve cardiometabolic risk factors in at-risk adults in the community and primary care setting [5–7]. Magkos et al. demonstrated substantial therapeutic effects of moderate weight loss at 5% among sedentary obese subjects on systolic blood pressure, plasma triglycerides and insulin sensitivity in multi-organ and β-cell function. Further increment in weight loss of 11 to 16% increases insulin sensitivity in muscle and improves adipose tissue biology [8]. This was evidenced in the Look AHEAD (Action for Health in Diabetes) trial whereby an average weight loss of 8.6% was achieved through an intensive lifestyle intervention consisting of home-based exercise with a target goal of 175 min of moderate intensity physical activity per week and caloric restriction. At 12-month post-intervention, the improvement was observed in mean HbA1c, blood pressures, lipid profiles and urine albumin/creatinine in the intervention group [9]. Follow-up data showed the benefits of intervention maintained in several markers including weight loss, HbA1c, systolic blood pressure and HDL-C among the participants of the intervention group for at least 4 years [10].

Although lifestyle intervention for weight loss has shown promising result, it is challenging to replicate it in a community setting. Furthermore, the impact of the intervention on cardiometabolic risk factors remains inconclusive due to variability of the studies such as enrolment criteria, intensity and modality of the intervention. At present, research assessing the effectiveness of weight management through lifestyle intervention on cardiometabolic health among overweight and obese adults with cardiometabolic risks in Malaysian community remains scarce. Previous studies generally focused on patients in clinic and individuals with comorbidities. My Body is Fit and Fabulous at Home (MyBFF@home) was the first community-based lifestyle intervention study among overweight and obese women who were at risk for cardiometabolic disease and lived in low socio-economic urban community in Klang Valley, Malaysia. This study aimed to evaluate the effect of weight loss lifestyle intervention on cardiometabolic markers at the completion of study from the baseline (Baseline to 12-month) as well as to see the changes by phase during the weight loss intervention phase (Baseline to 6-month) and weight loss maintenance phase (6-month to 12-month).

## Methods

### Study design and population

The protocol of the MyBFF@home study has been described previously [11]. MyBFF@home was a quasi-experimental study designed to examine the effects of 12-month lifestyle modification among housewives living in low-cost flats in Klang Valley, Malaysia. A total of 328 women were recruited (159: control group; 169: intervention group) during baseline visit. The first 6 months was the weight-loss intervention phase followed by 6 months maintenance phase. At baseline, 243 complete blood samples (114 for the control group and 129 for the intervention group) were collected after excluding incomplete measurement, failure to attend for blood test and non-fasting participants. Data for blood analyses were collected from the intervention and control groups at baseline, 6- and 12-month. All participants provided informed consent prior to entry into the study.

### Cardiometabolic markers assessment

**Fig. 1:**
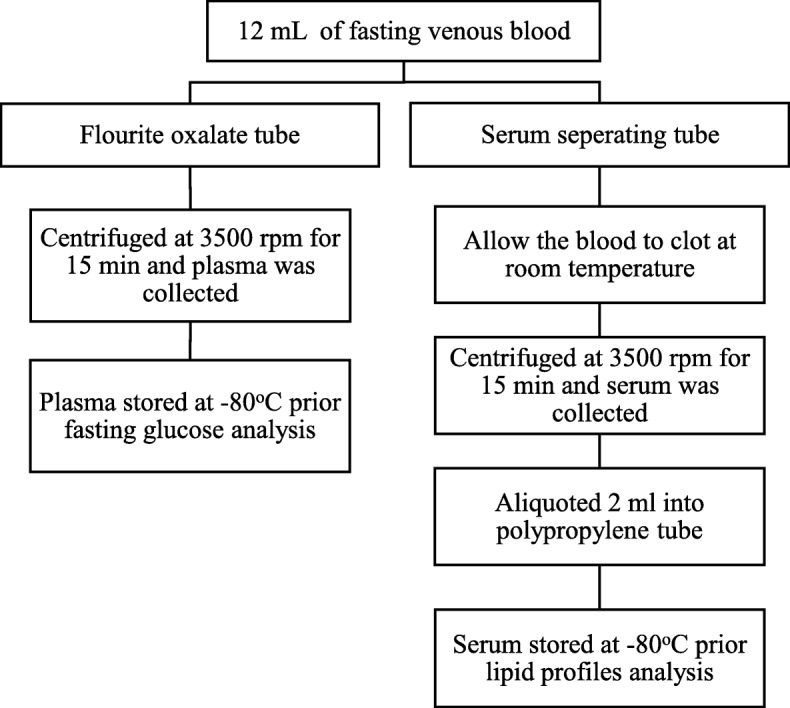
Flowchart of blood collection process

The participants were requested to fast for 10 h prior to the study visit. Blood was drawn by qualified nurses or medical assistant between 8 am to 10 am. The blood samples were transported on crushed ice to Institute for Medical Research (IMR) laboratory for processing. All samples were aliquoted into separate cryovials and stored at -80°C until further analysis. The blood collection process is summarized in Fig. 1. Fasting plasma glucose (FPG), total cholesterol (TC), triglycerides, high-density lipoprotein cholesterol (HDL-C) and low-density lipoprotein cholesterol (LDL-C) were analysed using Chemistry Analyser (CS-400 Dirui, China). The inter-assay coefficient of variant (CV) for glucose at 6.34 mmol/L and 16.06 mmol/L were 2.78 and 1.69% respectively, and for lipids ranged from 1.58 to 4.64%.

### Statistical analysis

All analyses were performed on an intention-to-treat basis; participants were analysed in the group that they were assigned to according to the phase of the study (ie. weight-loss intervention phase and maintenance phase). The distributions of baseline characteristics were examined for both control and intervention groups. Continuous variables were reported as means and standard deviation (SD) while categorical variables were described as frequency and percentage. Differences in baseline characteristics between study groups were compared using independent t-test for continuous variables and chi-square test for categorical variables. Paired samples t-test was used to assess the changes of cardiometabolic markers within the study groups while the difference of mean changes between groups was measured using independent t-test. Main effects of time x group for the overall intervention were assessed using general linear model (GLM) repeated measures ANOVA by controlling for age, education level, baseline weight and baseline of variables (as stated). The data were analysed using SPSS for Windows© version 22.0 (Chicago, IL, US). A *p*-value of < 0.05 was considered statistically significant (two-tailed).

## Results

### Baseline characteristics

**Fig. 2:**
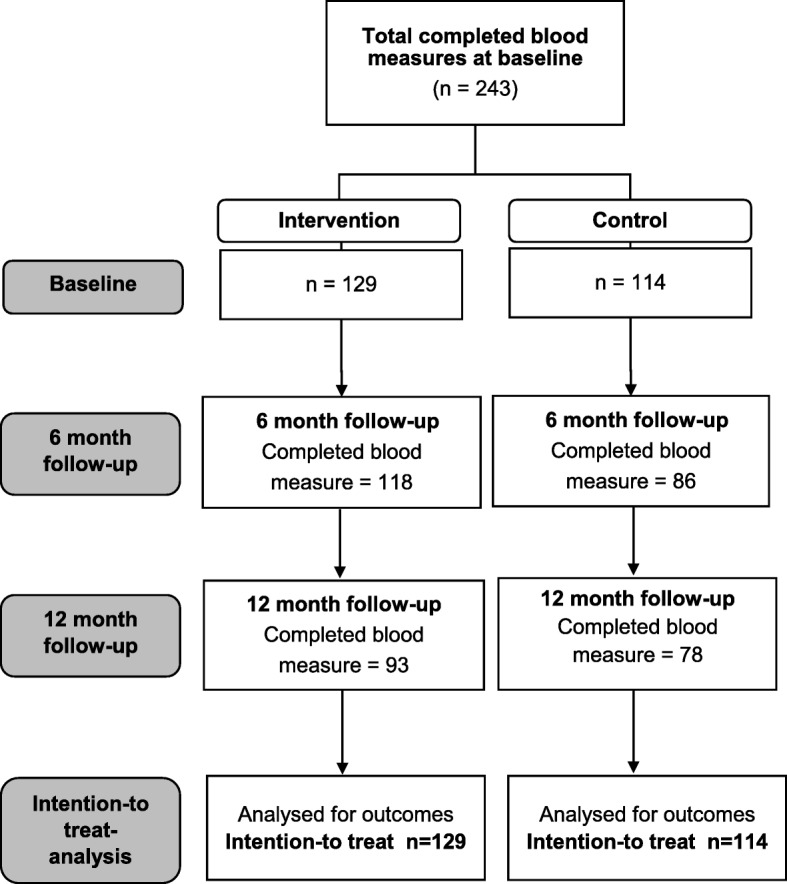
Study flow of participants in MyBFF@home for blood assessment

Figure 2 demonstrates the flow of participants for blood assessment throughout the study. Women who did not return for both 6- and 12-month visits were excluded from the analysis. The mean (SD) age of the participants were 41.98 (7.97) years and 42.31 (7.90) years in control and intervention group respectively. The BMI and cardiometabolic health characteristics of the intervention and control groups were similar at baseline except for HDL-C (Table 1). This supports the homogeneity of the groups at baseline. Most of the cardiometabolic markers at baseline were near borderline or at risk. Almost 50% of participants in both groups had family history of T2DM and hypertension and around 20% had family history of CVD. Although the participants included in this study were free from chronic diseases, the baseline characteristics suggest that this population is at risk of developing cardiometabolic diseases.

**Table 1:** Baseline anthropometric and cardiometabolic characteristics of participants in the MyBFF@home study

Characteristics	Control (*n* = 114)	Intervention (*n* = 129)	p-value^a^
Age, years	41.98 (7.97)	42.38 (7.89)	0.70
BMI, kg/m^2^	30.80 (4.10)	31.68 (4.14)	0.10
Family history of T2DM %(n)	47 (42.0)	62 (48.8)	0.29
Family history of hypertension %(n)	52 (46.4)	77 (60.2)	**0.03**
Family history of CVD %(n)	19 (17.3)	30 (23.6)	0.23
Cardiometabolic Risk Factors			
FPG, mmol/L	5.64 (1.03)	5.63 (1.45)	0.95
TC, mmol/L	5.70 (1.09)	5.59 (1.02)	0.44
LDL-C, mmol/L	4.59 (1.32)	4.44 (1.11)3	0.33
HDL-C, mmol/L	1.38 (0.23)	1.31 (0.23)	**0.02**
Triglycerides, mmol/L	1.35 (0.66)	1.35 (0.67)	1.00
LDL-C/HDL-C ratio	3.35 (0.91)	3.43 (0.86)	0.46
TC/HDL-C ratio	4.15 (0.71)	4.30 (0.71)	0.09

Note: Data are presented as mean (standard deviation) or % (number of participant)

Boldface indicates statistically significance

ª Determined with independent t-test for continuous variables and χ^2^ test for categorical variables, significant at p-value < 0.05

Abbreviations: *BMI* body mass index, *CVD* cardiovascular disease, *FPG* fasting plasma glucose, *TC* total cholesterol *LDL-C* low density lipoprotein-cholesterol, *HDL-C* high density lipoprotein-cholesterol

### Overall (baseline − 12 month) outcome

Most of the cardiometabolic markers showed improvements between baseline and 12-month follow-up in both study groups (Table 2). The intervention group showed within group improvement in FPG (− 0.30 mmol/L [95% CI: − 0.54 to − 0.06], *p* < 0.01) and LDL-C (− 0.39 mmol/L [95% CI: − 0.62 to − 0.17], *p* < 0.001). LDL-C was also reduced significantly within the control group at 12 month (− 0.54 mmol/L [95% CI: − 0.80 to − 0.27], *p* < 0.001). TC was reduced significantly in the intervention group with an average reduction of 0.26 mmol/L [95% CI: − 0.47 to − 0.06], *p* < 0.01) compared to the control (− 0.06 mmol/L [95% CI: − 0.28 to 0.17]). HDL-C however, showed significant reduction in the control group (− 0.09 mmol/L [95% CI: − 0.14 to − 0.04], *p* < 0.001) and the intervention group (− 0.05 mmol/L [95%CI: − 0.10 to −0.00], *p* < 0.05). The control group had significant increase in TC/HDL-C ratio (0.31 [95% CI: 0.08 to 0.54], *p* < 0.01) while no significant changes was found in the intervention group. Other variables showed no within group changes from baseline. The intervention effect was only found in TC (*p* < 0.05).

**Table 2:** Changes in cardiometabolic risk factors in control and intervention group overall (Baseline-12 month)

Outcome measures	Estimated mean difference (95% CI)^§^ (Baseline – 12 month)		Intervention effect (between group x Time)		
	Control	Intervention	F	*p*-value	Partial eta squared
FPG (mmol/L)	− 0.12	− 0.30**	0.894	0.410	0.008
	(− 0.39, 0.15)	**(− 0.54, − 0.06)**			
Triglycerides (mmol/L)	0.05	0.00	0.152	0.859	0.001
	(− 0.10, 0.19)	(− 0.18, 0.19)			
Total cholesterol (mmol/L)	− 0.06	**− 0.26**	3.131	0.046*	0.026
	(− 0.28, 0.17)	(− 0.47, − 0.06) **			
HDL-C (mmol/L) *	− 0.09***	− 0.05*	0.621	0.538	0.003
	**(− 0.14, − 0.04)**	**(− 0.10, − 0.00)**			
LDL-C (mmol/L)	− 0.54***	− 0.39***	0.543	0.582	0.005
	**(− 0.80, − 0.27)**	**(− 0.62, − 0.17)**			
LDL-C/HDL-C ratio	− 0.12	− 0.12	0.247	0.781	0.002
	(− 0.36, 0.12)	(− 0.31, 0.07)			
TC/HDL-C ratio^ψ^	0.31**	0.04	2.493	0.089	0.020
	**(0.08, 0.54)**	(− 0.14, 0.22)			

Notes: Data are presented as estimated mean differences (95% CI)

Boldface indicates statistical significance, *p < 0.05, **p < 0.01, ***p < 0.001

^§^Estimated mean differences obtained using repeated measures ANOVA adjusting for age, education level and baseline weight, significance at *p* < 0.05

^ψ^Adjusted for baseline values

Abbreviations: *CI* confidence interval, *FPG* fasting plasma glucose, *LDL-C* low density lipoprotein-cholesterol, *HDL-C* high density lipoprotein-cholesterol

### Weight-loss intervention phase outcome

Table 3 shows the changes of cardiometabolic markers within and between group during weight-loss intervention phase. A trend of reduction in FPG was observed in the intervention group at 6 months. Both groups showed improvement in TC with average reduction of 0.21 mmol/L ([95% CI: – 0.38 to − 0.05], *p* < 0.05) in the intervention group and by 0.27 mmol/L ([95% CI: − 0.42 to − 0.11], *p* < 0.01) in the control group. HDL-C decreased significantly in the control group (− 0.05 mmol/L [95% CI: − 0.10 to − 0.01] while, the level unchanged in the intervention group. However, no significant difference between-group was evidenced. Triglycerides increased in both groups but not clinically significant as the level was below the normal limit (< 1.7 mmol/L). A trend of reduction was found in LDL-C and TC/HDL-C ratio in both study groups. No significant changes were seen in LDL-C/HDL-C ratio in both study groups.

**Table 3:** Within- and between-group comparisons of cardiometabolic risk factors during weight-loss intervention phase

Outcome measures	Group	Baseline Mean (SD)	6 month Mean (SD)	Change within-group	*p*-value^a^	Mean Difference of change Between group	*p*-value^b^
				MD (95% CI)		MD (95% CI)	
FPG (mmol/L)	Control (n=114)	5.64 (1.03)	5.58 (1.28)	-0.05 (-0.25, 0.14)	0.569	0.06 (-0.23, 0.34)	0.692
	Intervention (n=129)	5.63 (1.45)	5.51 (0.96)	-0.11 (-0.32, 0.10)	0.287		
Triglycerides (mmol/L)	Control (n=114)	1.35 (0.66)	1.44 (0.72)	0.09 (-0.04, 0.22)	0.193	-0.01 (-0.20, 0.18)	0.906
	Intervention (n=129)	1.35 (0.67)	1.45 (0.63)	0.10 (-0.04, 0.23)	0.155		
Total cholesterol (mmol/L)	Control (n=114)	5.70 (1.09)	5.43 (0.97)	-0.27 (-0.42, -0.11)	0.001**	-0.06 (-0.28, 0.17)	0.620
	Intervention (n=129)	5.59 (1.02)	5.38 (0.85)	-0.21 (-0.38, -0.05)	0.012*		
HDL-C (mmol/L)	Control (n=114)	1.38 (0.23)	1.33 (0.23)	-0.05 (-0.10, -0.01)	0.011*	-0.05 (-0.11, 0.01)	0.103
	Intervention (n=129)	1.31 (0.23)	1.31 (0.25)	0.00 (-0.05, 0.04)	0.811		
LDL-C (mmol/L)	Control (n=114)	4.59 (1.32)	4.45 (1.09)	-0.14 (-0.37, 0.09)	0.223	-0.09 (-0.38, 0.19)	0.526
	Intervention (n=129)	4.44 (1.11)	4.39 (0.94)	-0.05 (-0.23, 0.13)	0.585		
LDL-C/HDL-C ratio	Control (n=114)	3.35 (0.91)	3.43 (0.91)	0.07 (-0.12, 0.25)	0.486	0.04 (-0.12, 0.28)	0.741
	Intervention (n=129)	3.43 (0.86)	3.43 (0.94)	0.03 (-0.13, 0.18)	0.748		
TC/HDL-C ratio	Control (n=114)	4.15 (0.71)	4.18 (0.79)	-0.02 (-0.14, 0.11)	0.82	0.05 (-0.17, 0.27)	0.642
	Intervention (n=129)	4.30 (0.70)	4.25 (0.88)	-0.07 (-0.24, 0.10)	0.432		

Notes: Boldface indicates statistically significance *p<0.05, **p<0.01

Mean differences within group and between group are in mean (95% CI), a negative change indicates a fall in average from baseline to 6 month

^a^Paired t- test (between baseline and 6-month measurement), significant at *p*<0.05

^b^Independent t-test, significant at *p*<0.05

Abbreviations: *SD* standard deviation, *MD* mean difference, *FPG* fasting plasma glucose, *LDL-C* Low-density lipoprotein-cholesterol, *HDL-C* High-density lipoprotein-cholesterol

### Maintenance phase outcome

During maintenance phase, most of favourable changes in the intervention group were not retained except for LDL-C (Table 4). LDL-C was further reduced in the intervention group by 0.34 mmol/L ([95% CI: – 0.48 to − 0.21], *p* < 0.001) and by 0.39 mmol/L ([95% CI: -0.54, − 0.23]) in the control group. HDL-C level however, was not maintained in the intervention group as reduction was seen during this phase. Whereas, FPG was significantly reduced in the intervention group (− 0.19 mmol/L [95% CI: -0.32 to − 0.06]) but the change was not significant when compared to control. A trend of reduction was seen in triglycerides of both groups but the changes were not significant. A significant between-group difference (*p* < 0.05) was observed in TC whereby, the intervention group retained TC reduction although not significant (− 0.05 mmol [95% CI: - 0.19 to 0.09]) while, the level increased significantly in the control group (0.22 mmol/L [95% CI: 0.06 to 0.38]). This attributed to significant increase in TC/HDL-C ratio in the control group (0.32 [95% CI: 0.15 to 0.50], *p* < 0.001) as compared to the intervention group (0.10 [95% CI: – 0.07 to 0.29]). Both groups showed significant reduction in LDL-C/HDL-C ratio during this phase due to significant reduction in LDL-C level.

**Table 4:** Within and between-group comparisons of cardiometabolic risk factors during maintenance phase

Outcome measures	Group	6 month Mean (SD)	12 month Mean (SD)	Change within-group	*p*-value^a^	Mean Difference of change Between group	*p*-value^b^
				MD (95% CI)		MD (95% CI)	
Fasting Glucose	Control	5.58 (1.28)	5.52 (1.49)	-0.06 (-0.24, 0.12)	0.485	0.13 (-0.09, 0.34)	0.254
	Intervention	5.51 (0.96)	5.32 (0.95)	-0.19 (-0.32, -0.06)	0.005**		
Triglycerides (mmol/L)	Control	1.44 (0.72)	1.39 (0.60)	-0.04 (-0.18, 0.09)	0.496	0.05 (0.06, 0.48)	0.584
	Intervention	1.45 (0.63)	1.35 (0.65)	-0.09 (-0.21, 0.03)	0.121		
Total cholesterol (mmol/L)	Control	5.43 (0.97)	5.64 (0.10)	0.22 (0.06, 0.38)	0.008**	0.27 (-0.10, 0.41)	0.013*
	Intervention	5.38 (0.85)	5.33 (0.96)	-0.05 (-0.19, 0.09)	0.488		
HDL-C (mmol/L)	Control	1.33 (0.23)	1.30 (0.24)	-0.03 (-0.07, 0.01)	0.195	0.02 (-0.04, 0.07)	0.520
	Intervention	1.31 (0.25)	1.26 (0.26)	-0.05 (-0.08, -0.01)	0.007**		
LDL-C (mmol/L)	Control	4.45 (1.09)	4.06 (0.96)	-0.39 (-0.54, -0.23)	< 0.001***	-0.04 (-0.25, 0.16)	0.690
	Intervention	4.39 (0.94)	4.05 (0.95)	-0.34 (-0.48, -0.21)	< 0.001***		
LDL-C:HDL-C ratio	Control	3.41 (0.91)	3.23 (0.96)	-0.18 (-0.32, -0.05)	0.009**	-0.04 (-0.22, 0.15)	0.683
	Intervention	3.46 (0.92)	3.31 (0.94)	-0.15 (-0.03, -0.02)	0.023*		
TC/HDL-C ratio	Control	4.14 (0.69)	4.46 (0.10)	0.32 (0.15, 0.50)	< 0.001***	0.22 (-0.03, 0.47)	0.089
	Intervention	4.24 (1.01)	4.35 (0.98)	0.11 (-0.07, 0.29)	0.234		

Notes: Boldface indicates statistically significance, *p<0.05, **p<0.01, ***p<0.001

Mean differences within group and between group are in mean (95% CI), a negative change indicates a fall in average from 6 month to 12 month

^a^Paired t- test (between 6 month and 12-month measurement)

^b^Independent t-test

Abbreviations: *SD* standard deviation, *MD* mean difference, *FPG* fasting plasma glucose, *LDL-C* Low density lipoprotein-cholesterol, *HDL-C* High density lipoprotein-cholesterol

## Discussion

This study demonstrates important evidence regarding the effect of a community-based lifestyle intervention on cardiometabolic health among overweight and obese women with cardiometabolic risks. The results showed that despite moderate approach (increased physical activity and diet control) provided in MyBFF@home study, a significant improvement in several cardiometabolic risk markers particularly TC was found in the intervention group over 12 months of study (intervention effect, *p* < 0.05). The results are consistent with other weight loss intervention trial in infertile obese women which reported modest weight loss (− 3.1 kg) with small yet significant improvement in systolic blood pressure (− 2.8 mmHg [95% CI: -5.0 to − 0.7]) and HOMA-IR (− 0.5 [95% CI: -0.8 to − 0.1]) in the intervention group compared to the control. Interestingly, the small changes resulted in halving the odds of metabolic syndrome (MetS) [12]. This supports the advocates on benefits of lifestyle modification in preventing or treating comorbidities among obese individuals independent of weight loss [13–15]. Ross and Janiszweski indicated that the benefits beyond weight loss are an important observation in obesity reduction programme in order to encourage the participants to continue with their attempts to change the behaviour as minimal or negligible changes of body weight may occur during the early stage of the programme [16].

Other lifestyle intervention conducted by Ibrahim et al. among prediabetes adults in Malaysian community (co-HELP) found that the participants in the co-HELP group loss a modest amount of weight (− 1.99 kg) with greater proportion of weight loss (> 5%) from initial body weight at 12 months. This confers the significant improvement in glycaemic control measurement including fasting glucose (− 0.40 mmol/L), 2-h post glucose (− 0.58 mmol/L) and HbA1c (− 0.24%) [17]. In our study, however, a lesser reduction in fasting glucose was found in the intervention group (− 0.30 mmol/L) at 12 months post-intervention which might be attributed by lower amount of weight loss compared to the mentioned study. The result was consistent with a study by Wing et al. that the amount of weight loss strongly related to the odds of having a clinically meaningful improvement in risk factors including glycaemic control measurement [18].

Our study demonstrates more reduction in TC within the intervention group at 6 month (− 0.21 mmol/L) and 12 month (− 0.26 mmol/L) compared to baseline level than other community-based lifestyle intervention among high risk individuals [19, 20]. Arsenault et al. [21] also found no improvement in lipoprotein lipid profiles when sedentary metabolically healthy overweight or obese women were subjected to 6-month exercise intervention. It was evidenced that significant reduction in LDL-C during maintenance phase, reduced the LDL-C/HDL-C ratio within both groups. While, an upsurge of TC level in the control group during the maintenance phase contributes to significant increase in TC/HDL-C ratio. The results suggest that the lifestyle intervention may regulate the level of lipid profiles and hence reduced the risk of CVD as LDL-C/HDL-C and TC/HDL-C ratios are better predictor for CVD risk in non-diabetes individuals than HDL-C, TC or LDL-C alone [22].

Despite improvement found within the intervention group particularly in FPG at 6 and 12 months as compared to baseline level, no significant between-group difference was detected in both of the time intervals. This can be explained by parallel improvement of the markers in the control group which consequently nullify the between-group effects. Apart from that, as explained earlier, modest weight loss (i.e. < 5% from the initial body weight, data not shown) in the intervention group might account for the small changes in the cardiometabolic markers. The results are comparable to other randomized control trial (RCT) where the effect of the intervention was underestimated due to similar positive changes in both intervention and control groups [20, 23]. Improvement in the control group is part of lifestyle intervention challenges as the trial requires the control group to be subjected to at least usual care treatment in which the placebo effect due to factors such as attention (Hawthorne effect), hope, structure and working alliance are difficult to be distinguished [24]. The follow-up session attended by the control group might also motivate them to lose weight and this was initially anticipated in this study. In addition, the positive changes of the control group suggest basic awareness of general health and subsequent weight monitoring might be sufficient to evoke change in the high-risk population.

MyBFF@home was unable to show effective sustainability of positive changes in cardiometabolic risks during the maintenance phase. This was anticipated as the participants in the intervention group are required to adopt healthy lifestyle independent personal counselling during this phase. Lack of social support could be one of the main barriers which can influence the adherence of women to active lifestyle. Majority of participants enjoyed group exercise during weight-loss intervention phase rather than doing it alone at home. Previous studies have shown the association between physical activity and social support particularly from family and friends which mediated by physical activity enjoyment [25, 26].

Another possible factor which hinders women to maintain the healthy lifestyle is time constraint. The participants are mostly middle-aged mothers with young children in which most of their times are dedicated for taking care of the children, family needs and house chores. Consequently, they find that they lack of time or too tired to exercise. The finding was also found in an in-depth study among Latina women where the participants reported no time as barrier to leisure-time physical activity and involvement in such activity may neglect their traditional responsibilities as mother and wife [27].

Apart from that, diet cost may become the main concern to low-household income populations in maintaining healthy nutrition. Higher diet cost is associated with higher quality of food such as vegetables, fruits and diet with lower calories from solid fat, added sugars and alcoholic beverages [28, 29]. As evidenced in a recent study among population in Selangor, Malaysia, a positive association was found between higher daily dietary cost and higher scores of Malaysian Healthy Eating Index particularly for components such as cereal products, vegetables and fruits [30]. In addition, Saleem et al. conducted a survey on cost assessment for healthy diet in several hypermarkets in Penang city, Malaysia. The authors revealed that healthy eating was unaffordable with an average per person for 1500 kcal menu of RM 845.50 and RM 1062.30 and 1437.60 for 2000 and 2500 kcal respectively each month [31] in which the expenses were comparable to living cost in Klang Valley.

Despite physical activity and dietary counselling intervention, the current study lack of cognitive behaviour therapy which has been identified as an important factor in successful long-term weight loss maintenance [32]. Weight regain problem could be minimised through behaviour therapy by addressing physiological obstacles, acquisition of and long-term adherence as well as effective weight-control behaviour [33]. Moreover, subsequent monitoring via individual meeting at least once a month and contact via phone or email resulted in successful maintenance of weight loss approximately 5–6% and other cardiometabolic risk over the 4 years as indicated by the previous study [10]. However, long-term support and monitoring are not practical when applied to the community as it demands more resources such as manpower and cost. Existing technology modes of communication, for example, smart phone and web-based application enhanced coaching and self-monitoring. The technology also promises potential benefits when incorporated in weight loss intervention as evidenced in several trials [20, 34]. Future studies should examine factors and treatment components which associated with sustainability of weight and other cardiometabolic health.

This study has several limitations. Intervention trial often recruited highly motivated individuals that are commonly more health conscious than the general population. It is a common bias that presents in conducting community-based intervention and would be difficult to be eliminated. Quasi-experimental design offers feasibility to conduct in community setting due to limited manpower and logistic issue but lack of randomization and may not sufficiently control the possibility of confounding factors. However, in our study, no compelling differences were detected in the baseline anthropometry and cardiometabolic risk markers between the control and intervention groups. Although the location of study groups in Klang Valley was separated geographically (control group at north and intervention group at south), there may be a chance of control contamination as the locations of each study groups are within 30 km radius. In addition, lower anticipated number of samples at post-intervention suggesting slightly under-powered to detect between-group differences. The results of this study should also be interpreted cautiously as it did not infer to the whole women population.

To our knowledge, this is the first study in Malaysia to evaluate the effectiveness of a community-based lifestyle intervention on cardiometabolic risk markers among overweight and obese women with cardiometabolic risks. The lifestyle intervention which promotes increased physical activity, healthy diet and self-monitoring confirm the potential value in cardiometabolic health changes observed at post-intervention, with possible sustainability at 12-month.

## Conclusion

A lifestyle intervention comprising moderate to light physical activity and diet control among overweight and obese women in the community could be effective to reduce cardiometabolic risk factors particularly fasting glucose, TC and HDL-C with favourable sustainability at 12-month. This study may provide an option to policy makers to enhance the current obesity intervention programme and subsequently improve health status of Malaysian women.

## Abbreviations

ANOVA: Analysis of variance
BMI: Body mass index
CI: Confidence interval
CV: Coefficient of variant
CVD: Cardiovascular disease
FPG: Fasting plasma glucose
GLM: General linear model
HDL-C: High-density lipoprotein cholesterol
LDL-C: Low-density lipoprotein cholesterol
MetS: Metabolic syndrome
NCD: Non-communicable diseases
RCT: Randomized control trial
SD: Standard deviation
T2DM: Type-2-diabetes mellitus.
TC: Total cholesterol
